# Perspectives, Measurability and Effects of Non-Contact Biofield-Based Practices: A Narrative Review of Quantitative Research

**DOI:** 10.3390/ijerph18126397

**Published:** 2021-06-13

**Authors:** Luís Carlos Matos, Jorge Pereira Machado, Fernando Jorge Monteiro, Henry Johannes Greten

**Affiliations:** 1 Faculdade de Engenharia da Universidade do Porto, 4200-465 Porto, Portugal; fjmont@fe.up.pt; 2 Centro de Biociências em Saúde Integrativa (CBSIn), Atlântico Business School, 4405-604 Vila Nova de Gaia, Portugal; jmachado@icbas.up.pt; 3 Centro Transdisciplinar de Estudos da Consciência (CTEC), Universidade Fernando Pessoa, 4249-004 Porto, Portugal; 4 Institute of Biomedical Sciences Abel Salazar (ICBAS), Universidade do Porto, 4050-313 Porto, Portugal; heidelbergschool@aol.com; 5 Instituto de Engenharia Biomédica (INEB), Universidade do Porto, 4200-135 Porto, Portugal; 6 Instituto de Investigação e Inovação em Saúde (i3S), Universidade do Porto, 4200-135 Porto, Portugal; 7 German Society of Traditional Chinese Medicine, 69126 Heidelberg, Germany

**Keywords:** biofield, nonlocality, consciousness, intention, healing

## Abstract

Practices such as “Reiki”, therapeutic touch, healing touch, and external “Qigong” have been regarded as some form of “energy medicine” or “biofield therapy”. The biofield concept has been studied and debated by researchers of distinct areas of expertise, and although the phenomenon was sometimes described as physically related to electromagnetics, other factors such as “subtle energy” and focused intention might be involved. These nonconventional practices integrate contact and non-contact techniques, and those dealing with so-called distant healing interventions are perhaps the most difficult to understand and accept. Practitioners describe these so-called nonlocal interventions as involving intentional factors and particular states of consciousness. With a spiritual mindset and a particular state of awareness, compassion is said to work out as a catalyst to produce physiological and physical changes through mechanisms that are still unknown. At the body level, these vegetative changes might be related to individual self-perception variations as part of the body neurovegetative feedback system of regulation. Further mechanisms are difficult to document and measure, and might be more accessible to research by using physical signal detectors, chemical dynamics methods, detectors using biological materials, detectors using living sensors, and detectors using the human body. The growing interest in these practices and the considerable amount of research exploring their effects and clinical applications encouraged this narrative review, which aims to provide an easy to consult partial overview of the history, theory and findings of quantitative research strategies exploring non-contact biofield-based practices. This work also aims to stimulate the reader’s mind with the raised hypotheses, catalyzing further research on the subject to confirm or deny the reported outcomes.

## 1. Introduction

The effects and mechanisms behind nonconventional healing practices have been studied over the last decades. The biofield, a field thought to exist within and around the body, is among researchers’ topics. To explore possible biofield effects and their hypothetical mechanisms, practitioners of traditional medicine and the so-called “energy healers” have been often involved in this research field.

Practices such as “Reiki”, therapeutic touch, healing touch, and “external Qigong” are regarded as some form of “energy medicine” or “biofield therapy”. Those are supported by therapists’ and patients’ perceptions and beliefs that some subtle, biological energy surrounds and permeates the body and is accessible for diagnostic and therapeutic interventions [1]. Energy medicine modalities have been categorized in the United States of America using two main classes: the veritable, which must be measurable using conventional technology, and the putative or subtle, which have not been definitively, scientifically measured [1,2,3]. This classification is no longer included on the website of the National Center for Complementary and Integrative Health (NCCIH); however, its foundation considers that these healing modalities are all based on the concept that human beings are infused with a subtle form of energy, often referred to as the biofield [4,5]. Indeed, some authors define energy medicine as a branch of integrative medicine that studies the science of therapeutic applications of subtle energies to assess and treat energetic imbalances, bringing the body’s systems back to homeostasis [6,7].

“Qigong” is a therapeutic tool of traditional Chinese medicine (TCM), which can be understood as a traditional vegetative biofeedback therapy consisting of concentrative motion and postures combined with breathing exercises and a particular mental state of “awareness”. Although frequently used as a self-regulation practice, it can also be part of a hetero-treatment performed by a qualified practitioner. In this case, it can be described as some sort of distance therapy (also called “qi” emission, “external qi” therapy or “bu qi”) in which the practitioner is thought to manipulate the patient’s “qi” by focusing on the energetic properties of the patients’ channels, collaterals, and points, as well as internal organs, from a distance of several inches, several feet, or even several miles away [8]. Sharing the eastern origin and some other particularities, “Reiki” is a practice derived from Japanese healing traditions and means literally “universal life force energy” [9]. In this system, the practitioner does not attempt to adjust the patient’s energy field or actively project energy into the patient’s body; neither is involved in assessing the patient’s energy field or actively attempting to reorganize or adjust it [10]. Instead, “Reiki” practitioners believe that they serve as a conduit and that healing energy arises from the practitioner’s hands and flows to where it is needed. It is in this way customized to the patient’s needs and condition [11]. “Reiki” may be given through a laying-on of hands or sent as a wish/prayer from any distance, holding the intention that whatever energy exchanges or transformations are needed, they should occur under a “higher intelligence” guidance [12]. Some aspects shared by these practices are also seen in the so-called “energy therapies” developed in the west by nurses, such as therapeutic touch and healing touch. These two practices are similar and might involve hands-on or -off approaches and the intention to balance the mind, body, and spirit to promote comfort and accelerate healing [12].

**Figure 1:**
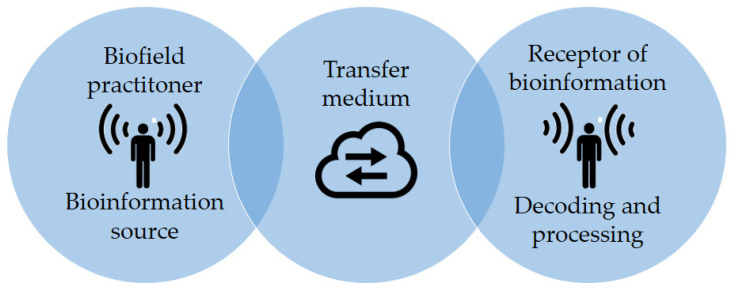
Hypothetical bioinformation transport mechanism established during a non-contact biofield intervention (adapted from [19]). Description: the biofield practitioner generates bioinformation signals carried in the transfer medium, reaching the receiving person whose homeostatic system decodes and physiologically process the information.

The mentioned complementary and alternative medicine (CAM) modalities see the human being as a holistic living system, exchanging energy and information with the environment. These CAM practices integrate the concept of “vital force” as the primary driving mechanism in health, pathogenesis, and healing. This “vital force” is often named as, for example, “Qi”, “Ki”, or “Prana” in Chinese, Japanese, and Hindu cultures. In a medical context, “Qi” can be described as a (neuro-)vegetative functional capacity of a tissue or an organ, which, in the case of TCM, is therapeutically influenced by reflex points in the body. However, some think that it might further be part of an innate biologic field of the body, in which bioinformation carried by tiny energy signals may trigger changes in molecular structures [13,14,15,16]. This sophisticated, dynamic, weak energy field might be involved in maintaining the whole organism’s integrity, regulating physiological and biochemical responses, and acting on development, healing, and regeneration [17,18]. Figure 1 illustrates the cybernetics of a hypothetical bioinformation transport mechanism established during a non-contact biofield intervention. In this system, the transfer medium might be understood as the medium carrying the signal generated by the practitioner, and the receptor is the patient receiving the treatment.

According to several authors, this phenomenon could be partially related to electromagnetics [20,21,22,23,24,25,26,27,28,29,30], to acoustic- and thermal-related effects [31,32,33,34], and possibly subtle energy fields, which, in some cases, seem to generate physical changes that are measurable with current technological methods, and related to health or disease patterns [13,35,36]. On the other hand, some practices appear to act in a manner described as nonlocal and unmediated, defying conventional scientific concepts [24,37], possibly compromising consciousness and transpersonal realms of being often associated with spirituality [17].

The concept of vital force and the spiritual framework of some of these practices are still not easy to integrate into the dominant biomedical paradigm [38]. Despite the inherent difficulties, the growing interest in spirituality and its effects, such as healthcare, has led science to attempt to define the subject to make it easier to quantify and validate [5].

Within many of these practices, intention, which could be defined as a directed thought to perform a determined action, plays an essential role in these processes. Some researchers even show data that may make it believable that intention may be powerful enough to change the physical reality, affecting inanimate objects and living things, from unicellular organisms to human beings [39,40,41,42,43].

**Figure 2:**
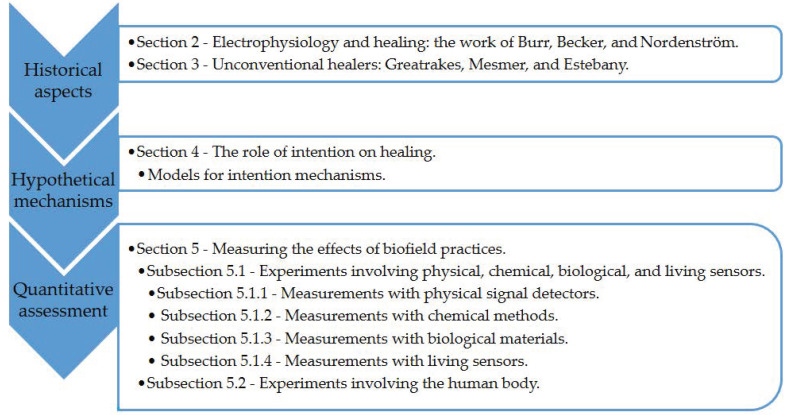
Sequence overview of the main topics covered in the present narrative review.

Covering the three main topics presented in Figure 2, the main goal of this narrative review is to provide an easy to consult partial overview of the history, theory and findings of quantitative research strategies exploring non-contact biofield-based practices. This work also aims to stimulate the reader’s mind with the raised hypotheses, catalyzing further research on the subject to confirm or deny the reported outcomes.

## 2. Electrophysiology and Healing: The Work of Burr, Becker, and Nordenström

Harold Saxton Burr, a professor at Yale Medical School, began studying the effects of energy fields in humans in 1932. According to Burr, all living organisms are surrounded by their energy fields, called Lifefields (L-fields), and changes in the L-field’s electric potential would lead to changes in the organism’s health state. Changes in environmental electromagnetic fields caused by the moon phases, sunspot activity, and thunderstorms affect L-fields significantly [29,44]. Indeed, some evidence points towards geomagnetic pulsation strongly entraining brain waves during meditation, making the mind quiet and dominated by geophysical rhythms. The holistic viewpoint emphasizes the connectedness among the body’s components and between the organism and its surroundings. In the case of meditation, this might be regarded as a connection with nature [35].

In the 1970s, Burr’s research on the development of the nervous system began a series of important but controversial studies about the role of electricity in development and disease. Some of his conclusions can be found in the book entitled “Blueprint for Immortality, The Electrical Patterns of Life”, also published under the title “The Fields of Life”. In this work, he postulates that the organism has a field as a whole, which embraces subsidiary or local fields, representing the organism’s parts. Changes in the subsidiary fields would be reflected in variations in the whole system’s energy flow. This electrodynamic field could serve as a signpost for various conditions [44]. In this sense, the human biofield might be influenced by the fields of nearby organisms, technology, earth, or even the cosmos.

Robert O. Becker, an orthopedic surgeon who developed research on electrophysiology, authored “The Body Electric. Electromagnetism and the Foundation of Life”. In this work, Becker claims that the current flows over the nerves’ perineural structures, traveling from the brain, that presents a higher electropositive potential to the body’s periphery. He also found a direct relationship between current, tissue growth, and healing processes by measuring the DC electrical potential and current involved in spontaneous and induced regeneration in various species such as salamanders, frogs, and rats. A remarkable level of limb regeneration in adult frogs was reported by applying a negative polarity current to the post-amputation stump, and that fractures of the long bones in frogs demonstrated a negative polarity along with apparent dedifferentiation of the erythrocytes in the fracture hematoma, which later formed the bone “callus” responsible for healing the fracture [45]. His results show that bone can generate potentials by the piezoelectric effect and that the natural repair of bone fractures can be stimulated by electric current [13,20].

Björn Nordenström was Chairman Emeritus of the Department of Radiology at the Karolinska Institute. He also chaired the Nobel Assembly Committee that selects the Nobel Laureate in Physiology and Medicine and has performed remarkable research on cancer treatment. He developed the electrical circulatory system theory, where the body is composed of “biologically closed electrical circuits”. In this model, the body’s electrical communication system can be compared to a battery in which the separation of oppositely charged ions drives the circuit. He noticed that when the tissue is damaged by injury or malignant growth, there is a build-up of positively charged ions in the affected area, whereas the adjacent healthy tissue is negatively charged. In his book entitled “Exploring BCEC-Systems (Biologically Closed Electric Circuits)”, he points out that ancient Oriental philosophy and approaches are related to his theory, considering that “qi” is analogous to the energy flow through his electrical circulatory system and that yin and yang deal with negative and positive charges, respectively [29,46].

## 3. Unconventional Healers: Greatrakes, Mesmer, and Estebany

The earliest recorded medical investigations on unconventional healing interventions began in 1665 when Dr. Thomas Sydenham and other renowned physicians documented the ability of Valentine Greatrakes, the famous “Irish Stroaker”, to eliminate pain, cure the King’s evil (scrofula), reduce swelling, and alleviate a wide range of other disorders, by lightly stroking his hands either on or proximate to the physical body. Greatrakes was wondrously successful in healing thousands of persons from all across Britain and Ireland. His practices were observed by the best-known intellectuals, philosophers, theologians, and physicians at that time, many of them writing pamphlets and letters detailing their thoughts about the stroker’s amazing cures [47]. One of them was Sir Robert Boyle, one of the founders of modern physics and chemistry and the discoverer of what came to be known as Boyle’s law. Boyle suggested that “perhaps some salubrious streams of spirits” were induced from Greatrakes hands into the patient’s body [48].

One century later, Franz Anton Mesmer, a Viennese physician living in Paris in 1778, postulated that some “fluidium” existed as a force of nature subjected to physics laws. His doctoral dissertation, “De Planetarium Influxu” was submitted to the Faculty of Medicine of the University of Vienna in 1766. This work was the theoretical basis for the model known as “gravitas animalis” or “magnetismus animalis”. It explored hypnosis and a primitive description of the cyclical activity in the biosphere, electricity, magnetism, and even a variant on Newton’s recently described gravity [49]. Mesmer began using magnets for healing, and his patients frequently noticed “unusual currents” coursing through their bodies. This phenomenon could be reproduced by only passing his hands above the patient’s body. When the scientific community was invited to witness his practice in treating some diseases, it was considered ridiculous, closely resembling the laying on of the hands of Jesus and other religious figures [35].

Mesmer stated that the ability to cure comes from a universal force called “animal magnetism”, which he could concentrate on and transmit to patients. The controversy around his practices led Louis XIV to appoint a committee, including Benjamin Franklin, Antoine Lavoisier, and Joseph Guillotine, to investigate his activities’ validity. This group concluded that Mesmer’s claims were false, and the positive results were simply due to his ability to manipulate the patients’ imagination [50]. As a result, hypnosis’s historical roots are frequently associated with Mesmer’s animal magnetism technique [51]. Despite the controversy, Mesmer’s work marks psychophysical self-regulation and hypnosis and psychosomatic medicine development. The rise of mind–body issues came due to the animal magnetism theory [49].

Oskar Estebany, a Hungarian army colonel in the mid-1930s, noticed that the horses he groomed recovered from illnesses faster than those treated by others [20]. A study conducted by Grad et al. [52] showed that the healing of surgical wounds in mice was enhanced by placing Estebany’s hands near the cage. These results were statistically significant and successfully replicated, suggesting no placebo effect [52]. Other studies were made with the cooperation of Estebany, who also showed the ability to speed up the growth of barley plants and reactivate ultraviolet-damaged samples of trypsin, a stomach enzyme, in much the same way as a magnetic field, even though no magnetic field could be detected near his body with the instruments of that era [20,53,54,55,56,57,58].

## 4. The Role of Intention on Healing

Several authors have studied the physical effects of intention. The results of those experiments could even indicate that human intentions would remotely influence cellular function, microbial growth, the growth of tumors in animals, the germination of seeds, the growth of plants, the healing of surgical wounds in animals, the kinetics of biochemical reactions, and have significant effects on nonbiological settings [41,59]. These results raised the thesis that humans might remotely influence each other’s physiology through the simple act of staring and thinking, even when the distant individual is unaware that the effort is being made [47].

The healing process established between a healer and a patient might involve entanglement and some form of mutual awareness. The system is sealed by the intention of healing and the need to be healed [60]. In this process, the healer, with a focused sense of inner quietude, establishes an intention to help, strengthened by empathic compassion or loving kindness directed toward the patient [61,62]. This connection enhances the sense of meaning of both the healer and patient and might resemble some aspects of meaning-centered psychotherapy, which has been proved to have beneficial effects in treating mood and anxiety disorders [63].

Usually, the sense of inner quietude experienced by the practitioner requires a self-process of centering, similar to meditation, whereby he turns his everyday conscious attention inward to a place of stillness to focus his mind and emotions. As the practitioner focuses on his intent, a particular state of awareness is experienced with a sense of wholeness [64]. In this scenario, it is thought that compassionate healing intentions might induce measurable effects on the target, the empathic connection between the healer and the patient being an essential factor in the process [47].

This phenomenon’s nonlocality character is often related to the concept of entanglement, where an element cannot be fully described without considering one or more additional elements [65]. In the case of distant healing, the healer who directs his thought or intent, and the patient, who receives it, create a single system, even physically separated. Here, entanglement is used to describe a connection between two elements, even though separated across space [1].

Studies involving neuron-to-neuron, brain-to-brain, and person-to-person connections have been carried out. The work of Pizzi et al. [66] showed that by stimulating a group of human neurons with a laser beam, a different group of neurons placed at a distance exhibited similar changes, although the two groups were entirely shielded from each other [66]. Other studies point towards changes in the alpha rhythms of twin’s brains when one of the subjects, away from his brother, closes his eyes. Changes in patients’ brains, detected by functional magnetic resonance imaging (fMRI), are also detected when healers focus their distant intention to heal [59].

Some experiments suggest that water’s physical properties, such as the cooling rate, molecular bonding reflected by infrared spectra alterations, Raman spectroscopy, scattered laser light, and the pH level, may be influenced by intention [30,39,41,43,67,68,69,70].

Radin et al. [39] conducted a double-blinded experiment to evaluate the effects of distant intention on water crystal formation. After a blinded assessment performed by 100 independent judges, the authors concluded that crystals from the “treated” water were given higher aesthetic appeal scores than those from control (*p* = 0.001) [39]. In a previous study, Emoto [68] found that positive intentions tend to produce symmetric, well-formed, aesthetically pleasing crystals, in contrast to the asymmetric, poorly formed, unattractive crystals produced by negative intentions [68].

Although Emoto’s findings are not consensual, some authors suggest that this phenomenon might be related to water memory and the imprinting of specific electromagnetic frequencies at a structural level, which may cause distinct physical changes with effects in processes as in crystallization [15,67]. According to Mayor [10], a frequency might be retained in a water coherence domain if its protons’ magnetic resonance is synchronized with the applied frequency. The protons would generate their internal magnetic field to satisfy the resonance conditions [10].

### Models for Intention Mechanisms

The hypothetical mechanisms through which intention has a physical effect on the target are still under debate. Some authors suggest that this process may be related to the ultraweak photon emission naturally occurring in all living organisms [71]. The biophoton emission is considered an excellent communication system, triggering, almost instantaneously, signals between the emitter and the receptor. Research suggests that the human body emits 10 to 103 photons s^−1^⋅cm^−2^ and that health and disease patterns could be related to the degree of emission [72].

Biophotons are information carriers, mediating cell-to-cell communication in several microbes, plants, and animals. The nervous system can emit biophotons continuously, and the inherent bioelectrical activities may affect biophotonic emission. Therefore, biophotons can mediate the transmission and processing of nerve signals and encode neural signals through intensity and frequency [73].

There is evidence that intracerebral biophoton activity changes are related to consciousness and phosphene phenomena. Some studies have shown that when subjects sitting in the dark imagined a white light, reliable and significant photon emission increases at 15 cm from the right cerebral hemisphere were noticed, strongly correlated with specific frequencies of electroencephalographic activity [74]. These results may imply that imagination, often related to intentional thought in remote healing processes, correlates with ultraweak photon emission and brain activity.

Instrumentation such as the photomultiplier detector and charge-coupled device (CCD) cameras helps measure and differentiate health and disease patterns and identify light channels in the body that might regulate energy and information transfer between different parts [75]. Biophoton research may promote the development of objective diagnosis tools, providing experimental biophysical support to particular TCM theory on meridians, as well as to TCM concepts such as the “eight principles” (yin/yang, interior/exterior, cold/heat, and deficiency/excess) or even certain aspects of the vegetative effects of the “qi” [73,76]. Moreover, some anatomical locations, such as the palm of the hands and the forehead, which are thought to be strong emitting areas, are typically associated with acupuncture and “Qigong” essential points.

**Figure 3:**
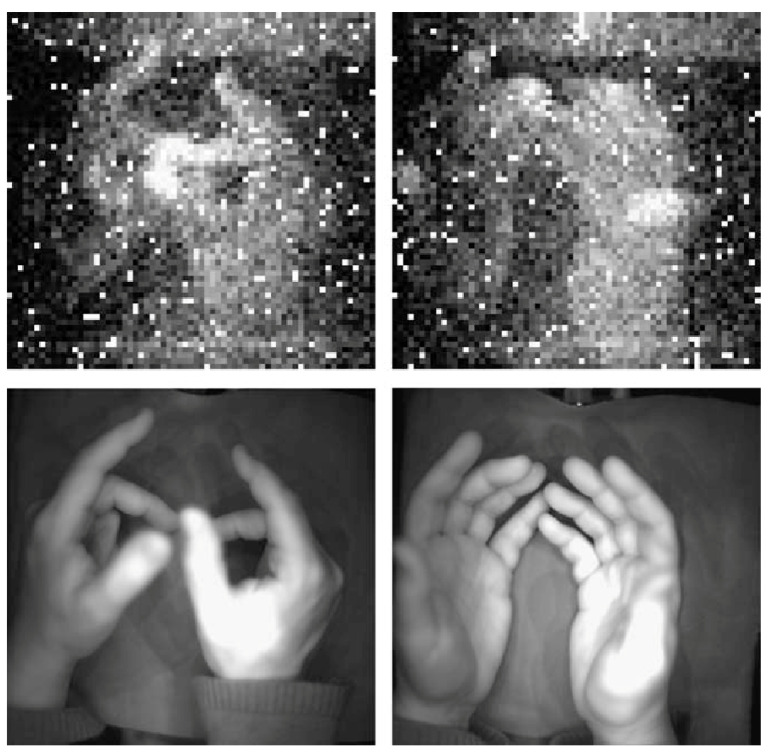
Top images are 10-min exposures taken in total darkness using 20 × 20 binning with a Princeton Instruments VersArray 1300B camera (Teledyne Princeton Instruments, New Jersey, USA) cooled to −100 °C. Bottom images are 10 ms exposures taken with white-light illumination. Reprinted from [77]. The top and bottom images are captures of the same situation/subject with different techniques. The top images are the pixelized representation of biophoton emissions obtained with a cooled, low-noise CCD camera in total darkness (biophoton emission: fingertips > palms > back of the hands). The bottom images were obtained with white-light illumination.

In 2005, Creath and Schwartz published a paper reviewing some of their findings while developing biophoton imaging instrumentation for monitoring biofields around living organisms to access their health state quantitatively and gather information about the healing process. The main findings of these authors reveal that plants ‘‘glow in the dark’’ and that biophoton emission imaging provides information about metabolic functioning, the health state of the organism, and that the phenomenon seems to be reactive to the intention of a healer. Figure 3 shows images of Creath’s hands by using a cooled, highly sensitive silicon CCD camera with 10-min exposures in total darkness. The bottom two images were taken in white light, while the top two images are biophoton images taken in total darkness [77].

**Figure 4:**
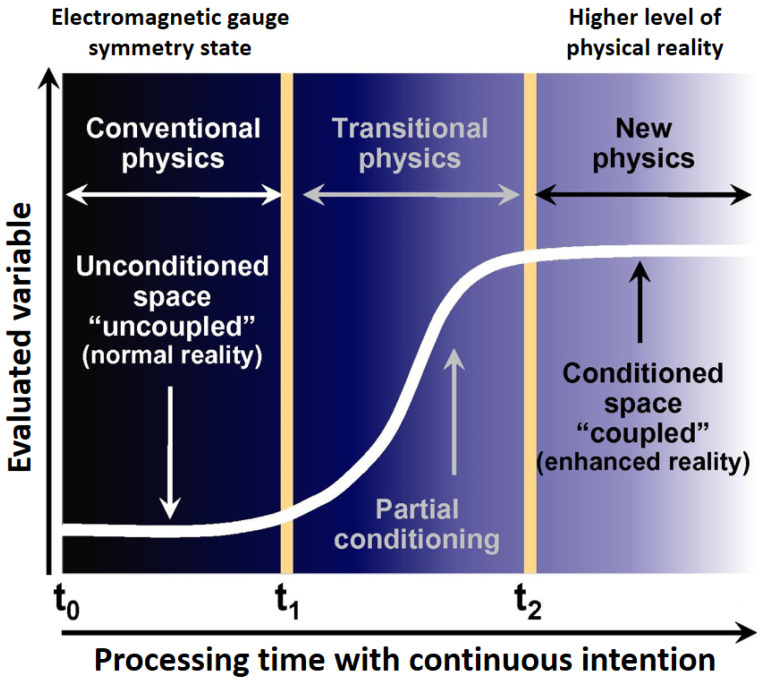
The time-evolution pattern of a*n* intention target experiment. Reprinted with permission from [78]. Copyright 2011 Elsevier (Amsterdam, The Netherlands) and Copyright Clearance Center (Danvers, MA, USA). Time-dependent conditioning of a space in which a variable is measured, for example, water pH, under a continuous intention. As time goes on (time zero – t_0_; time one – t_1_; time two – t_2_), the electromagnetic gauge symmetry state of the space changes from uncoupled to coupled and consequently to a higher level of physical reality (higher electromagnetic gauge symmetry state).

According to William A. Tiller, Professor Emeritus of the Department of Material Sciences and Engineering at the University of Stanford, space might become changed or “conditioned” when submitted to a continuous intentional stimulus. Tiller and his colleagues hypothesized that the fundamental symmetry state could be altered by activating the indwelling consciousness of the space to a higher level of physical reality, thus changing the electromagnetic gauge symmetry state of that space, which in turn allows the human intentions to change the properties of materials significantly. Tiller even suggests the existence of two unique levels of physical reality, the uncoupled state, where the electric, magnetic dipole, molecular, and atomic states are dominant, and the coupled state that appears to function throughout the physical space, interpenetrating the vacuum and the electric, magnetic dipole, atomic and molecular states. Figure 4 illustrates Tiller’s model for the conditioning of space under the influence of intention.

According to this model, human consciousness and intention can promote these two categories’ interactions. Considering that the coupled state would have a higher thermodynamic free energy per unit volume than the uncoupled state, it can perform work of any kind on the uncoupled electric, atomic, molecular subsystem [67,79,80,81,82,83,84].

## 5. Measuring the Effects of Biofield Practices

The biofield hypothesis and related healing practices require the existence of a measurable “healing energy” that, whether produced by a device or projected from the human body, has a particular frequency or set of frequencies that stimulates the repair of one or more tissues. The cascade of activities initiated by such signals may provide essential information to cells and tissues and open channels for information flow that coordinates both prevention and repair processes [35,85].

Besides other published studies on the medical applications of “Qigong” and emitting “qi” to humans, animals, cell cultures, and plants [31,85,86,87], an analytical review on this subject was published in 2004 by Kevin W. Chen, Professor at the University of Medicine and Dentistry of New Jersey. According to this review involving studies conducted in China in the last decades, the assessment of the “external qi” effects was made considering five different categories of detectors: (1) physical signal detectors, (2) chemical dynamics methods, (3) detectors using biological materials, (4) detectors using living sensors, and (5) detectors using the human body [32]. This categorization seems to be a reasonable approach to aggregate and describe the diversity of studies on the topic and will be used in the present narrative review.

### 5.1. Experiments Involving Physical, Chemical, Biological and Living Sensors

Clinical and preclinical studies in real-world populations are needed to provide a complete picture of health, illness, and treatment based on these practices. However, in a preliminary stage, animals, plants, biomolecules, tissue, and cell cultures are good research models because there is no expectation or belief, nor are they affected by psychosocial factors [88]. The following subsections present many studies assessing biofield effects with physical signal detectors, chemical methods, biological materials, and complex living organisms used as sensors.

#### 5.1.1. Measurements with Physical Signal Detectors

Instrumentation to measure physical parameters is required to access biofields practices’ mechanisms. Objective measurements are needed to ‘calibrate’ both sources and receptors and to standardize the procedure. This approach is essential to know if a negative result is given to the receptor or the healer.

The existence of electromagnetic fields within and around the body and how these fields affect biological systems is often related to the body’s movement of charged particles. Considering that biological systems radiate and absorb electromagnetic frequencies, external or environmental radiation might also induce body changes [85]. Research has shown that humans can change these fields’ properties [21,25,89]. Table 1 presents the main findings of selected studies and reviews that explored the effects of non-contact biofield practices assessed with physical signal detectors.

In a study conducted by Moga et al. [25] on the effects of hands-on healing and distant healing of mice with induced tumors, the authors found anomalous magnetic field activity during the intervention. The peak-to-peak variations of magnetic field (MF) oscillations were higher than baseline and had a symmetrical wave-like appearance, resembling discrete packets of sinusoidal waveforms, decreasing and increasing in frequency over time. Using Fast Fourier transform analysis, the authors found that in the beginning, the frequency was 20 to 30 Hz, slowing to 8 to 9 Hz and then to less than 1 Hz, and from this point, the wave reversed and increased again. The MF oscillation ranged from 1 to 8 mG in strength with a duration of 60 to 120 s. Another study carried out by the same author using a Hall-type gaussmeter close to healer-client pairs during healing touch sessions showed low-frequency magnetic field oscillations during 24 of 26 healing touch sessions and 14 of 16 guided progressive relaxation sessions. The magnetic field oscillations (peak-to-peak) amplitude was significantly greater during the healing touch session and post-session periods than in the pre-session period. Peak-peak showed no significant change across the guided progressive relaxation periods. Large-amplitude magnetic field oscillations > 1.0 milliGauss during healing touch were associated with healer/client qualitative reports of emotional release and clearing of the biofield [90].

Unusual large body voltages have also been reported during therapeutic touch practitioners’ treatments. Considerable anomalous body potential surges ranging from −4 V to −190 V in therapeutic intervention and from −4 V to −221 V, lasting from 0.5 to 12.5 s in meditation, were measured at the ear lobe with an electrometer. On average, these electrical signals were 103 times larger than psychophysiological galvanic skin potential changes associated with emotional responses, 105 times larger than electrocardiographic voltages, and 106 times larger than electroencephalographic voltages [91]. Comparable results were obtained by Tiller et al. [92] under well-controlled conditions. In this study, an experienced therapeutic touch practitioner produced voltage changes between −20 V and −80 V from baseline, with similar durations [92].

As previously mentioned in the introduction, the “external qi” therapy is part of the medical “Qigong” tradition. Although most “Qigong” practice tries to reach the empty mind or nothingness state, some practitioners have shown the ability to use their mind or intent to guide the “qi” to the desired place. Some “Qigong” healers are thought to direct their “qi” outward to help unhealthy individuals break the “qi” blockage or balance the “qi” system [8].

Over the last 30 years, researchers have been trying to measure the external “qi” (“wai qi”) during “Qigong” healing. Although none have shown the primary nature or mechanism behind the phenomenon, some attempts point towards measurable effects. Moreover, some results cannot be explained by psychosocial effects or known biological processes, but do share the following aspects: the manifestation of the external “qi” is only noticed when a well-trained “Qigong” practitioner enters into a particular mental state of awareness, and it does not manifest in a normal state of mind; it can affect distant objects and produce measurable signals; it might affect a specific target not producing changes in the objects nearby the practitioner, where his intention is not focused [32].

Researchers have used different instruments to measure the related effects because the phenomenon was often thought to be connected to light, electricity, heat, sound, and magnetism. These include magnetometers, voltmeters, photometers, gamma radiation counters, sound equipment, and gas discharge visualization. Measurable changes have been reported, such as: modified far-infrared radiation emitted at a distance of 50 cm from the palm of a “Qigong” practitioner, with variations in intensity as high as 80% at a frequency of 0.3 Hz, which contrast with the control group (non-practitioner) that showed almost no difference in intensity; changes in the body surface temperature measured with infrared sensors during “Qigong” practice, both in the “Qigong” practitioner and patient; changes in the signal emitted by Ge (germanium) micro-pressure detectors that were placed at the distances of 0.5, 1, 1.5, and 2 m from the “Qigong” practitioner who emitted “external qi” toward the target through two of his fingers; significant increases in wavelength to above 10 mm during the “external qi” emission, measured by a mm-wave radiation meter placed 20 to 40 cm from the Qigong practitioner; changes in the magnetic field during “qi” emission, in well-controlled conditions in a zero-magnetism laboratory, with signals reaching 105 nT and contrasting with the weak ones emitted by non-practitioners; infrasonic sound pressure measured in acupoints during “qi” emission by experienced practitioners (48.8 to 54.7 dB) higher than those emitted by the control group (40.6 to 43.6 dB), reproducible in other studies where “Qigong” masters were able to increase these signals above 70 dB with a dominant peak frequency in the range 8 Hz to 12.5 Hz, which coincides with the frequencies of electroencephalography (EEG) alpha waves [26,27,31,32].

Seto et al. [26] studied the magnetic field strength adjacent to the palms of “Qigong” practitioners’ hands during external “qi” emission. In 3 out of 37 cases, the authors observed a 4 to 10 Hz oscillation in the magnetic field, with a peak-to-peak magnetic field strength of 2 to 4 mG [26]. Such results represent an increase in strength 1000-fold higher than usual, as the human body radiates magnetic fields of less than 10 to 6 gauss [95]. Similar magnetic field strengths during healing practices have been reported by other authors [25]. Although this phenomenon’s origin is not yet understood, the physical effects seem measurable [93,96,97].

Emerging concepts in physics such as nonlocality and entanglement might provide a theoretical basis for these observations. There is also a view of consciousness and relativistic quantum physics that attributes an essential role to the mind, which might be relevant in this phenomenon [13,17,59,98].

An excellent example of this paradigm is the Yan Xin “Qigong” phenomenon. Yan Xin is a reputed chief physician, “Qigong” Master, and researcher with close relations to some Chinese Government Institutions, such as the Chinese National Natural Science Foundation, who supported some of his research projects. Since the 1980s, several scientists from leading universities and research institutes, such as Tsinghua University (Beijing, China), the Chinese Academy of Sciences (Beijing, China), Harvard University (Cambridge, MA, USA), University of California (Berkeley, CA, USA), and Oklahoma University (Norman, OK, USA), have been using scientific methods and protocols to investigate the various effects of the “external qi” emitted by Yan Xin. The results are challenging and suggest that the “qi” of Yan Xin can be projected out of the body and affect physical substances and objects. Some relevant findings are the detection of the external “qi” effect by thermoluminescent dosimeters and liquid crystals; the ability to change the conditions of chemical reactions; the interaction with matter from molecular to nuclear levels, specifically the molecular structure of liquid water and other water solutions measured by Raman spectroscopy; the effects on the half-life of radioactive isotope 241Am, measured by gamma-ray spectrometry and solid-state nuclear track detector, after “qi” emissions from a distance that ranged from 3 m to 10,000 km [93,96,97].

Although many of the reported outcomes seem to show changes in the assessed variables, other studies detected no changes. A good example is a study conducted by Baldwin et al. [94] to determine whether “Reiki” practice increases the electromagnetic field strength from the heart and hands of Reiki practitioners using a superconducting quantum interference device (SQUID). These authors found no electromagnetic field intensities greater than 3 pT in any of the recordings, leading to the conclusion that practicing Reiki does not appear to routinely produce high-intensity electromagnetic fields from the heart or hands [94].

#### 5.1.2. Measurements with Chemical Methods

Some authors have noticed that the “external qi” can change some chemical reactions’ dynamics. It seems that “Qigong” practitioners can use the “external qi” to change the activity of glucose oxidase (GOD), accelerating the kinetics of the following reaction:

(1) $$C_{6}H_{12}O_{6}+O_{2}\overset{\mathrm{GOD}}{\Rightarrow }C_{6}H_{12}O_{7}+H_{2}O_{2}$$

The hydrogen peroxide produced in the previous reaction reacted with luminol, and the resulting fluorescence was measured with a photoelectron detector. Ren et al. [99] found that “Qigong” practitioners can increase the reaction velocity by 400% from a distance ranging between 2 and 10 m, while 5% is the regular standard deviation for the reaction under the same conditions [99].

Under normal conditions, hydrogen peroxide gradually decomposes into water and oxygen; however, the decomposition was faster under the “external qi” influence [32,100].

Under the effect of intense light, normal hexane and bromide produce bromohexane and hydrogen bromide. Although this reaction requires intense light, researchers have shown that the reaction occurs without light under the influence of the “external qi” [32,101].

Biofield practices were also shown to enhance sucrose crystals’ growth from supersaturated solutions previously submitted to thermal treatment. Teixeira et al. [102] suggest that biofield interventions act synergistically on these mechanisms and that water may be the target, the vehicle, and the medium for these processes [102].

#### 5.1.3. Measurements with Biological Materials

The mechanisms through which biofield practices exert an effect on the biological domain are not clear-cut. Some authors suggest that connective tissue plays an essential role in this process. The living matrix is regarded as the continuous molecular fabric of the organism, consisting of fascia, other connective tissues, extracellular matrices, integrins, cytoskeletons, nuclear matrices, and DNA [103]. That continuum might be granted by integrins connecting the cytoskeleton of every cell with neighboring cells and the surrounding extracellular connective tissue and connections across the nuclear envelope, which join the cytoskeletal matrix with the nuclear matrix. In this picture, fascia could play an essential role in physiological regulation due to its connections to the interior of cells and cell nuclei and its electronic conduction properties. Collagen embedded in a soft polymer gel known as the “ground substance” is thought to store electrical charge as collagen acts as a semiconductor and the matrissomes as capacitors. Once each collagen molecule has a helical shell of water molecules intimately associated with it and orientated in an electric field, the coherent phase-correlated system formed by water might explain the sensitivity to resonant interactions with signals such as the ones generated by biofield practices [10]. The biological effects of subtle energies involved in biofield practices could be mediated and enhanced by cellular amplification and stochastic resonance, as shown in Ross Adey and A. R. Liboff’s research [104,105,106,107]. Thus, besides being a protective shield, the cell wall might work as an amplifier for these stimuli. The subsequent cascade of signals can act on various intracellular processes, among them calcium ion transport and enzymatic activity, which, by themselves, can trigger changes at the physiological regulatory level [13,29,108,109,110]. Table 2 presents the main findings of selected studies and reviews that explored the effects of non-contact biofield practices on biological materials.

Changes in the structure of biomolecules under the influence of “external qi” were reported by Chu et al. [111]. While studying the effects of “external qi” emitted by a Chinese “Qigong” master on poly-d-glutamic acid sodium salt and RNA conformations, these authors found that all poly-d-glutamic acid sodium salt samples presented some changes in circular dichroism spectra measured with a 62DS spectropolarimeter. A total of 67% of those samples had significant changes (more than three standard errors), and a ratio of ellipticity change of 1 to 10% with a maximum change of over 10.9%. No significant changes were noticed on RNA conformation [111,112]. “External qi” was shown to have effects on liver cancer cells (BEL-7402) and lung cancer cell culture (SPC-A), on blood plasma cAMP, on the structure and pharmaceutical characteristics of vitamin C, on the DNA synthesis and living cycles of liver cancer cells, on the phase behavior of dipalmitoylphosphatidylcholine (DPPC) liposomes, on the microstructure of *Escherichia coli* and tumor cells in mice. Some authors have shown that the “external qi” also enables the growth of Fab protein crystals, has an inhibitory effect on the growth of hepatitis B virus in vitro, and an inhibitory effect on the growth of human liver cancer cells (BEL-7402). Changes in the electric potential in fresh tree leaves, the acceleration of the germination and growth of plant seeds, including rice, wheat, peas, beans, peanuts, and flowers, and physical mutagen activity of a species of Streptomyces have also been reported [31,32,87,96,97,113].

The effects of “Reiki” on biological materials have been studied in controlled conditions. Kent et al. [114] found that a “Reiki” practitioner with more than 30 years of experience was able to significantly increase the photon emission of mice intervertebral disc cells compared to sham (*p* < 0.05). Real-time PCR (RT PCR) showed an increase in collagen II and aggrecan (*p* < 0.05) after the “Reiki” treatment, which might indicate an enhancement of the healing cascade in cells [114].

Trivedi et al. [121] tested the effects of a noncontact biofield treatment over in vitro cultures of human glioblastoma cells and healthy brain cells compared to untreated controls. The practitioner, seated at a short distance from the cells, was instructed to deliver the treatment outside an acrylic environmental chamber that enclosed the cells and microscope. Cell behavior was followed by time-lapse videomicroscopy, and data analysis was performed by a technician blinded to the treatment. The authors found an exponential increase in the cell death rate (41%) after treatment compared to control, which remained relatively constant throughout the 20-h testing period. The treated healthy brain cultured cells showed a significant reduction (64%) of cell death rate, suggesting a protective effect of the biofield treatment [121].

Similar results were found by Yan et al. [118] while studying Yan Xin “Qigong’s” effect over small-cell lung cancer cells. The results show that “external qi” induces cell death and gene expression alterations, promoting apoptosis and inhibiting proliferation, migration, and glucose metabolism in small-cell lung cancer cells. This phenomenon may induce an anticancer effect by modulating gene expression to facilitate cancer cell apoptosis while repressing proliferation, metastasis, and glucose metabolism [118]. In 2013, the same author published a study showing that “external qi” has a strong cytotoxic effect on colorectal cancer HT-29 cells, suggesting that it can be potentially used for colorectal cancer treatment directly or indirectly via carriers [119]. Additionally, external “qi” seems to exert anti-lung cancer effects while inhibiting signaling pathways that are important for non-small lung cancer cell survival and metastasis [122].

Although the previous studies point towards measurable positive effects over cultured cells, this behavior does not seem consensual or reproducible. In a study conducted by Yount et al. [115], “Qigong” practitioners directed healing intentions toward normal brain cell cultures for 20 min from a minimum distance of 10 cm. A standard colony-forming efficiency assay measured cell proliferation. The authors found a trend of increased cell proliferation in “Qigong”-treated samples showing, on average more colony formation than sham samples (*p* = 0.036); however, in a replication study (60 experiments), no significant difference between Qigong-treated samples and sham samples was observed (*p* = 0.465) [115]. Using time-lapse videomicroscopy to access potential glioblastoma cellular responsiveness to “Johrei” from a short distance, Taft et al. [116] found no evidence of a reproducible cellular response to “Johrei” treatment, concluding that the cell death and proliferation rates of cultured human cancer cells do not appear responsive to this biofield treatment from a short distance [116]. To clarify if the treatment duration (dose) or the distance between the biofield practitioner and the target cells play a role in the expression of the results, Yount et al. [123] invited an internationally recognized biofield practitioner to treat human glioblastoma cultured cells in well-controlled conditions. The authors found that the three mock/control experiments’ cell-viability ratios were all close to zero, while those involving biofield treatments of increasing dosage appeared to be monotonically decreasing. Thus, the most significant cancer-cell inhibition was observed when the practitioner was closest and delivered the highest dose. Further experiments from different distances, including replicating one of the first set of tests, failed to produce significant differences, leading the authors to consider the data inconclusive because of the inability to reproduce the cellular response in a replicate experiment [123].

The role of intention in the manifestation of the “external qi” was studied in a controlled experiment using the proliferation of *Escherichia coli* as a target. Opposite intentions of promoting proliferation and killing during the application of the “qi” therapy resulted in higher and lower optical density, respectively, after incubation compared to control [117]. The manifestation of opposite intentions during “external qi” emission might result in distinct physical effects, such as increased or decreased infrared radiation and temperature near the practitioner’s hands. Bacterial growth has been used as a parameter to measure biofield effects. Lucchetti et al. [120] studied the effects of Spiritist “passe” and laying on of hands (LOH) in four settings: no intention, intention to inhibit bacterial growth, intention to promote growth, and influence of a negative factor. Those experiments, carried out with control, showed that under the intention to inhibit bacterial growth condition, statistically significant differences were found between the Spiritist “passe” and “no LOH” groups (*p* = 0.002 after 48 h, and *p* = 0.008 after one week) and also between the Spiritist “passe” and “LOH” groups (*p* = 0.005 after 48 h, and *p* = 0.009 after one week). No statistically significant difference was found in the experiments with no intention, with an intention to promote growth, and when LOH was performed under the influence of a negative factor (watching a war scene of a movie) [120].

Still exploring the biochemical effects of intention and “external qi” therapy on human fibroblast FS-4, researchers found that facilitating “qi” caused a 1.8% increase of cell growth in 24 h, 10 to 15% increase in DNA synthesis and 3 to 5% increase in protein synthesis of the cell in 2 h, while inhibiting “qi” caused a 6% decrease of cell growth in 24 h, 20 to 23% decrease of DNA synthesis and 35 to 48% of protein synthesis in 2 h. Additionally, the respiration rate of boar sperm increased by 12.5 to 13.0% after receiving facilitated “qi” for 5 min and decreased by 45 to 48% after receiving inhibiting “qi” for 2 min [87].

#### 5.1.4. Measurements with Living Sensors

This group includes research involving complex living organisms that resemble humans’ biological characteristics, such as mice, rats, flies, rabbits, fish, dogs, toads, and pigs.

Baldwin et al. [124] studied “Reiki’s” effects on noise-induced microvascular damage in rats. The authors found that rats submitted to 15 min of noncontact “Reiki” before 30 min of noise every day for three weeks presented a reduced amount of mast cell degranulation and less extension of microvasculature leakage. Sham “Reiki” was delivered by students who were naive to this therapy by mimicking the “Reiki” practitioner’s hand positions. Rats submitted to sham “Reiki” showed no significant reduction in stress-related biomarkers [124].

Mice are often chosen as an animal model in research, mainly when the studies deal with cancer therapeutics. The possible effects of “external qi” on cancer growth, metastasis, and survival time of mice transplanted with cancer cells have been studied for decades. For example, in a study from the 1990s, tumor models were formed in 114 mice by transplanting U27 or MO4 cells into their subcutaneous tissues. The results showed that the average tumor volume in the “Qigong” group was lower than that in the control group (2.2 vs. 6.3 cm^3^; *p* < 0.001), the metastatic rate was lower (1/16 vs. 6/15; *p* < 0.05), and the average survival time was longer (35.4 vs. 30.5 days; *p* < 0.01) [125]. In another study from the same decade, the anti-tumor efficacy of “external qi” emission from a “Qigong” healer on transplanted hepatic cancer in mice was evaluated. Thirty mice injected with transplanted hepatic cancer cells were randomly assigned into one of three groups: (1) the control group (no-treatment), (2) the imitation group (sham treatment with imitation of “Qigong” movements), and (3) the “Qigong” group. The “Qigong” and sham treatment included four sessions of “qi” emission towards the mouse cage at a distance of 8 to 10 cm for 10 min. The results from three repeated experiments were similar. The true treated group’s tumor growth inhibitory rates were 70.3%, 79.7%, and 78.7%, respectively (*p* < 0.0001), higher than the control group. The inhibitory rates of the imitation-treated group were 9.5%, 2.6%, and 2.5%, respectively (*p* > 0.05). Electron microscopy showed that the morphological alterations in mice treated with “Qigong” included decreased cell volume of most cancer cells, nuclear condensation, nuclear fragmentation, decreased nucleus and cytoplasm ratio swollen mitochondria with poorly organized mitochondrial cristae, some vacuolated, and many apoptotic bodies in extracellular space. These results indicate that the “qi” emitted from a well-trained “Qigong” healer could inhibit transplanted hepatocarcinoma growth in mice [32,126].

Even with the growing interest in this thematic, there is still limited research examining if humans could inhibit cancer cells’ proliferation and suppress tumor growth by modifying inflammation and the immune system. Recently, a study carried out by Yang et al. [127] suggests that exposure to purported biofields from a human may be capable of suppressing tumor growth, which might in part be mediated through modification of the tumor microenvironment immune function and anti-inflammatory activity in our mouse lung tumor model. Indeed, these authors found that human NSCLC A549 lung cancer cells exposed to a purported healer showed reduced viability and downregulation of pAkt. The authors further observed that the experimental exposure slowed the growth of Lewis mouse lung carcinoma, evidenced by significantly smaller tumor volume in the experimental mice (274.3 ± 188.9 mm^3^) than that of control mice (740.5 ± 460.2 mm^3^; *p* < 0.05). Exposure to the experimental condition markedly reduced tumoral expression of pS6, a cytosolic marker of cell proliferation, by 45% compared with the control group. The reversed-phase proteomic array suggested that the experimental exposure downregulated the PD-L1 expression in the tumor tissues. Similarly, serum cytokines, especially MCP-1, were significantly reduced in the experimental group (*p* < 0.05). Furthermore, TILs profiling showed that CD8^+^/CD4^+^ immune cell population was increased almost twofold in the experimental condition, whereas the number of intratumoral CD25^+^/CD4^+^ (T-reg cells) and CD68^+^ macrophages were 84% and 33%, respectively, lower than that of the control group [127].

A subsequent publication involving the same experimental setup showed that the biofield treatment did not inhibit tumor growth but enhanced cancer cell death, in part mediated by modifying the tumor microenvironment and stemness of tumor cells. Tumors exposed to biofield treatment had a significantly higher percentage of necrosis (24.4 ± 6.8%) compared with control (6.5 ± 2.7%; *p* < 0.02), and cleaved caspase-3 positive cells were almost 2.3-fold higher (*p* < 0.05). Similarly, tumor-infiltrating lymphocytes profiling showed that the CD8^+^/CD45^+^ immune cell population was significantly increased 2.7-fold when exposed to biofield (*p* < 0.01), whereas the number of intratumoral FoxP3^+^/CD4^+^ (T-reg cells) was 30.4% lower than control (*p* = 0.01), leading to a significant 3.1-fold increase in the ratio of CD8^+^/T-reg cells (*p* < 0.01). Additionally, there was a 51% lower level of strongly stained CD68^+^ cells (*p* < 0.01), 57.9% lower level of F4/80high/CD206^+^ (M2 macrophages; *p* < 0.02) and a significant 1.8-fold increase of the ratio of M1/M2 macrophages (*p* < 0.02). Furthermore, biofield treatment resulted in a 15% reduction of stem cell marker CD44 and a significant 33% reduction of SOX2 compared with control (*p* < 0.02). The experimental group also engaged in almost 50% less movement throughout the session than the control [128].

A controlled study involving mice injected with 66cl4 mammary carcinoma cells collaborated with an internationally known healer who provided the subjects’ biofield treatment. Each treatment lasted approximately five minutes and consisted of the practitioner sitting two feet away from the mice on a low bench with the tops of their cages open (four mice per cage) and performing a mental energy transmission technique. The authors reported no significant differences in weight, tumor size, or metastasis in subjects treated with biofield therapy. Nevertheless, significant effects were found in the immune responses of mice treated after the cell injection. The biofield treatment significantly reduced the percentage of CD4^+^CD44loCD25^+^ and percentage of CD8^+^ cells, elevated by cancer in the lymph nodes, to control levels determined by FACS analysis. Only CD11b^+^ macrophages were increased with cancer in the spleen, and the biofield therapy significantly reduced them. Of 11 cytokines elevated by cancer, only interferon-γ, interleukin-1, monokine induced by interferon-γ, interleukin-2, and macrophage inflammatory protein-2 were significantly reduced to control levels with human biofield therapy. No additional effects were found in mice treated before the cell injection. The authors concluded that human biofield therapy had no significant effect on tumor size or metastasis but produced significant effects on immune responses in the down-regulation of specific lymphocytes and serum cytokines in a mouse breast cancer model [129]. Previously, the same authors reported similar results but involving therapeutic touch practitioners. Human biofield therapy had no significant effect on tumor size in that study but produced significant effects on metastasis and immune responses compared to mock-treated mice. Therapeutic touch treatment was proved to reduce IL-1-a, MIG, IL-1b, and MIP-2 to control/vehicle levels and reduce specific splenic lymphocyte subsets [130]. Using a randomized and controlled study design, other authors have shown that the biofield treatment provided by a certified healing touch practitioner was able to decrease cortisol levels in mice injected with murine mammary carcinoma 4T1 [131].

Eighteen pigs (weighing 8.5 to 12.5 kg) were deliberately injured by cutting off their T-vertebrae, removing external fat, and damaging their spinal cords with Allen’s method to produce the standard model of spinal paralysis. The injured pigs were randomly assigned into three groups: group A (*n* = 6) was treated by “external qi” 12 h after the injury, had three treatments a day for the first week, then had two treatments daily for 84 days. Group B (*n* = 6) started the “Qigong treatment” seven days after the injury, with two treatments a day for 84 days. Group C (*n* = 6), the control, had no treatment and was observed for a total of 90 days. All pigs were equally fed during the experimental period. At the end of the study, all pigs in group A could walk around freely, and two of them could run and jump, showing different degrees of recovery of their nervous functions. In group B, all except one pig could stand by themselves, and one could run around. None of the pigs could stand up in group C, and only two had some avoidance response to stimulation [132]. Similar findings were reported when the experiment was conducted with dogs using the same design and the same “Qigong intervention” [32,133].

### 5.2. Experiments Involving the Human Body

The physiological changes in the healer to initiate healing, the mechanisms that allow the person, animal, cells, or other systems to receive and process the healing, and the nature of the transmission between the healer and the receptor are essential topics to explore in research. The small sample size of some studies, the reduced amount of randomized controlled trials, and the inexistence of standardized methods to calibrate biofield therapists are limitations in this field of study [134]. Despite these limitations, evidence suggests a significant improvement in wellbeing compared to control subjects under circumstances that do not seem susceptible to placebo and expectancy effects [135,136]. Table 3 presents the main findings of selected studies and reviews that explored the effects of non-contact biofield practices on the human body.

During a biofield-based intervention based on practices such as “Reiki” and healing touch, the practitioner might use contact, non-contact, or a combination of both techniques. Whatever the approach used, biofield practices are often related to an evident reduction of stress patterns, as shown by Reeve et al. [145], while evaluating the effects of healing touch as an intervention in treating posttraumatic stress disorder. These authors found a significant mean reduction of symptom severity of about 18.11 points in the experimental group, comparable to a change of 5.57 points for the control group. Those exposed to biofield treatment reported a range of positive physical and psychological effects [145]. Most of these biofield interventions share several aspects related to the practitioner mindset, focus, and modus operandi. The primary outcomes might be associated with a compensation of stress’s adverse effects via sympathetic activation of recipients’ left-anterior cerebral cortex, as shown in the study carried out by Pike et al. [143]. The results of that study point towards an enhancement of the left-anterior activation of the cerebral cortex relative to placebo and no-treatment controls (as indicated by significantly higher and significantly positive alpha asymmetry index scores) during the first 100 s of treatment, indicating a higher overall reduction in state anxiety relative to baseline measures [143]. Other stress-related physiological variables are also responsive to biofield treatments, as Lee et al. [137] show. In that crossover study, “external qi” therapy was proved to induce significant changes accessed by encephalography and circulating cortisol concentrations in the real intervention compared to the placebo control. Subjects reported improved emotions of satisfaction, relaxation, and calmness during the real intervention compared to placebo treatment [137]. “External qi” therapy positively affected sympathovagal function compared to placebo, reducing the heart rate and increasing the heart rate variability [139,140]. These physiological mechanisms might be common to studies reporting beneficial effects of biofield practices in burnout [146], anxiety, and depression [147,148,149,150].

The previous studies support some of the findings of a systematic review by Jain et al. [4] to synthesize the best evidence examining whether such modalities positively affect health and reduce disease symptoms. That review included 66 clinical studies encompassing several biofield therapies in different patient populations. The authors found strong evidence for reducing pain intensity in pain populations and moderate evidence of reducing pain intensity in hospitalized and cancer populations. Additionally, moderate evidence points to decreased negative behavioral symptoms in dementia and anxiety in hospitalized populations. This study points to reducing fatigue, improved quality of life in cancer patients, and decreased cardiovascular patients’ anxiety [4].

Although the results of some studies seem promising and capable of leaving a clue about the possible involved mechanisms, other studies with good experimental designs did not seem to reveal any significant effects of these practices. To understand the underlying physiological mechanisms of how “Reiki” might have therapeutic effects on subjective measures of stress, pain, relaxation, and depression/anxiety, Bat [151] conducted a pilot randomized, double-blinded, and placebo-controlled study accessing the effects of this biofield modality on heart rate, diastolic and systolic blood pressure, body temperature, and stress levels. The changes in pre- and post-treatment measurements for each outcome measure were analyzed through an analysis of variance (ANOVA) post-hoc multiple comparison test, which found no statistically significant difference between any of the groups. Even though the significance level for comparing “Reiki” and sham groups for heart rate was 0.053, a definitive conclusion cannot be made based on this pilot study alone, demanding a study with a larger sample size [151].

While some researchers explore the effects of biofield practices on physiological parameters, others look for structural changes in the body, such as tumors or warts, to evaluate these structures’ remission effect. For example, the single-blinded, assessor-blinded, placebo-controlled, randomized trial conducted by Gaillard et al. [144] aimed to evaluate if one session of non-touch biofield therapy with an experienced practitioner could treat warts of the hands and feet in adults. The authors found that no original wart had disappeared three weeks after intervention (0/64). As well, there were no significant differences between the experimental and placebo groups concerning wart disappearance three weeks after intervention (*p* = 0.49), nor at six weeks (*p* = 0.40); however, a reduction in size at week 3 was more promising for biofield therapy, but was not significant (*p* = 0.27). This study had a short follow-up time to measure the clinical outcome, making it harder to verify the hypothesis [144].

Chronic pain is a common condition in the elderly population. In 2005, 43 elderly adults with chronic pain were randomly assigned either to an intervention or a general care group. The intervention group was given four weeks of “qi” therapy, whereas the control group was given standard care. Compared to the control group, the “qi” therapy participants experienced improvements in mood and psychological variables over the four-week program. Pain and psychological benefits remained significantly improved after two weeks of follow-up. These findings suggest that “qi” therapy may help the elderly to cope with pain and associated mood disturbances [141] and are in agreement with the systematic review conducted by Lee et al. [86]. In that review, 141 potentially relevant randomized clinical trials (RCT) on the effects of “external Qigong” in pain conditions were identified; however, only five RCTs were included due to the study excluding criteria. All RCTs on external “Qigong” demonstrated more a significant pain reduction in “Qigong” groups than control groups, including general care for treating chronic pain [86].

More recently, studies involving pain populations have been published, constituting a robust body of evidence on these practices’ beneficial effects. Some conditions with positive outcomes include multifactorial pain [86,141,152,153], carpal tunnel syndrome [154], palliative care [155], fibromyalgia [156,157], knee arthroplasty, postoperative pain [158], knee osteoarthritis [159,160], pain during hospitalization for cesarean [161], and premenstrual symptoms, including pain [138].

Biofield therapies have also been studied in cancer populations. Cases of improvement and the effectiveness of “qi” therapy in managing advanced cancer symptoms have been reported. For example, in one study case, the subject had beneficial effects on pain, vomiting, dyspnoea, fatigue, anorexia, insomnia, daily activity, and psychological calm. These improvements were maintained over the two-week follow-up phase. After the first “qi” therapy session, the patient discontinued medication and could sit by himself, and after the fourth session, the patient was able to walk and use the toilet without assistance [162]. In two different case studies, the authors found that after 20 min of “qi” therapy, both patients experienced improvements in mood and alertness and reduced pain, anxiety, depression, discomfort, and fatigue, on both the first and last days of the interventions. Furthermore, the scores recorded on the last day for most symptoms were improved compared to the first day [163].

The cytotoxic activity of natural killer (NK) cells is positively correlated to survival in cervical cancer patients. These cells target and lyse tumor cells and perform tumor cell surveillance. In 2010, Lutgendorf et al. conducted a study to assess how healing touch may affect immunity, depression, and treatment side effects in patients receiving chemoradiation for cervical cancer. The authors observed a marked reduction of NK cell activity in both the relaxation and usual care groups, in contrast to a mild decrease of NK cell activity in the healing touch group. The healing touch group showed a more significant decrease in two indicators of depressed mood compared to the other groups of patients [164].

Jain et al. [165] evaluated the effect of a biofield healing intervention over fatigue and cortisol variability in breast cancer survivors. The authors compared the intervention with mock treatment and wait-list groups and found statistically significant reductions in total fatigue for the biofield group compared to the wait-list group. Additionally, biofield treatment significantly increased diurnal cortisol variability to healthy levels compared to the mock treatment and wait-list groups [165]. Even though the reported improvements in breast cancer patients, this tendency is not always observed, which, in some cases, might be due to sampling size effects and study design. For example, Cohen et al. [142] carried out a study to determine whether “external qi” therapy could shrink breast cancer tumors and improve women’s quality of life with pathologically confirmed breast cancer awaiting surgery. The authors found no clinical changes in tumor measurements from pre- to post- “external qi” therapy, no suggestion of a change in tumor size by physical breast examination, and no changes in quality of life [142].

## 6. Conclusions

The human biofield is a challenging concept that is not fully compatible with the dominant biomedical paradigm. The mechanisms behind this phenomenon are still unclear or unknown. However, some experimental results and clinical applications support rather than deny its existence, thus justifying further studies about its nature and action mechanism. Nevertheless, there is still some stigma related to this subject and much anecdotal evidence lacking scientific methodology, contributing to some resistance to accepting this concept into mainstream scientific research. The literature on the subject is still weak, some references are not readily available to consult, and some are only written in Chinese. Evidence-based approaches are required to assess the effectiveness of these practices and concepts. In this context, well-designed studies with proper controls and standard procedures are required to ensure quality and reproducibility. The fulfillment of these requirements is a challenge to research, as a high number of variables might have an impact on the study, such as the practitioners’ background, the number of practitioners involved in the study, the type of biofield therapy, the duration of the focused intention intervention, and the frequency of biofield therapy during the experimental period. This field of research requires in-depth scrutiny and clarification, as there are still many question marks. Considering the healer a crucial element in the healing process and the supposed source of driving force and biofield activation, what kind of physiological changes occur in these subjects? Often measured biomarkers include electroencephalography (EEG) and heart rate variability (HRV); however, EEG changes seem inconsistent and not specific to biofield therapies. Baldwin and Hammerschlag [166] found that HRV results suggest an activated physiology for reconnective healing, Bruyere healing, and Hawaiian healing, but no changes were detected for “Reiki” and therapeutic touch [166]. Are these results due to the biofield practice itself or due to the practitioner’s experience and particular abilities? Here, a parameterization effort similar to the one seen in TCM diagnosis and practices [167] is imperative to “calibrate” the healers and healing process. Recently, Connor et al. [168] reported a set of portable and cost-effective measures that scientists could use to determine the competence of “energy practitioners” so that qualified practitioners could be selected to improve research accuracy. Those measures involved the use of a triaxial extra low-frequency magnetic field meter, data logging multimeter, radio frequency (RF) field spectrum analyzer, acoustimeter, broadcast frequency counter, digital pH meter, digital total dissolved solids (TDS) meter, gas discharge visualization (GDV) and physiology suite including heart rate variability, galvanic skin response, respiration, electromyography (EMG), electrocardiography (EKG), temperature and blood volume pulse [168]. The expected results from proper experimental designs may contribute to the debate and help understand the possible effects and involved mechanisms.

Future studies should be planned in an integrated manner, involving academia and research institutions, the healthcare community, and therapists associations to explore aspects of fundamental sciences and the clinical applicability of these practices. Further research on this subject should include the effects on electrophysiology and homeostasis, the assessment of changes in physiological and physical variables, the test of the nonlocality character of the intervention and the eventual entanglement within the system, and the demystification of philosophical terminology such as the circulation of “qi” or “prana” in meridians or “nadis”, in TCM and Ayurvedic medicine, respectively; the “energetic” fields of “dantians”, known as “chakras” in Ayurvedic medicine; or even the layers of the human energetic field, commonly referred to as the person’s aura. A particular emphasis should be given to research exploring the effects of intention and its relation to the biofield. For example, TCM holds that “xue” (similar to blood) is moved by the “qi” and guided by the “yi”, the intention of “shen” (mind). Therefore, the activation of the “qi flow” depends on the practitioner’s particular mental state of awareness, which triggers the manifestation of vegetative physiological changes, such as the increase of the microcirculation and changes in the electrical potential of the skin. As these changes have been measured during “Qigong” practice [169], how do they relate to the biofield? Measurements of biophotonic emission from the human body, particularly the hands, during “Qigong” or other biofield-based practice will be helpful to understand whether this physiological response has power enough to trigger changes, for example, in biological material placed in the vicinity of the practitioner. Additionally, measurements of the magnetic field properties in well-controlled conditions during the interventions might be valuable to check if eventual changes are assessed systematically and if they support space-conditioning hypotheses associated with intentional factors. Here, the use of water as a sensor and the evaluation of its properties by vibrational spectroscopy might help determine if the intervention has effects, not only in the experimental space but also in target and non-target materials within that space. Few studies have recently been published in this field [43,70,170], demanding more investment and research to confirm or deny the reported outcomes.

## Figures and Tables

**Table 1:** Research exploring the effects of non-contact biofield practices assessed with physical signal detectors.

Study	Main Findings
Green et al. [91]	Anomalous body potential surges ranging from −4 V to −190 V in therapeutic intervention and from −4 V to −221 V, lasting from 0.5 to 12.5 s in meditation, were measured at the ear lobe of therapeutic touch practitioners.
Seto et al. [26]	Three subjects exhibited a strong bio-magnetic field of 2 to 4 m Gauss in a frequency range of 4 to 10 Hz near the palms during “qi” emission.
Sancier&Hu [31]	Increased infrasonic sound signals emitted by “Qigong” masters were above 70 dB with a dominant peak frequency in the range 8 Hz to 12.5 Hz, which coincides with the frequencies of EEG alpha waves.
Tiller et al. [92]	Anomalous body potential surges ranging from −20 V to −80 V lasting from 0.5 to 12.5 s were measured at the ear lobe of therapeutic touch practitioners.
Hisamitsu et al. [27]	An extremely strong magnetic field was emitted from two subjects during “Qigong” breathing practice.
Waechter&Sergio [21]	The fields projected by the hands of “Qigong” masters affected the magnetic field power at specific frequencies near the detector.
Yan et al. [93]	The “external qi” emitted by Dr. Yan interacted with thermoluminescence dosimeters (TLD detectors) and generated responses similar to that induced by a mixed field of gamma rays and neutrons.
Chen [32]	Modified far-infrared radiation was emitted at a distance of 50 cm from the palm of a “Qigong” practitioner, with variations in intensity as high as 80% at a frequency of 0.3 Hz, which contrast with the control group (non-practitioner) that showed almost no difference in intensity.
	Changes in the body surface temperature were measured with infrared sensors during “Qigong” practice, both in the “Qigong” practitioner and patient.
	Changes were detected in the signal emitted by Ge (germanium) micro-pressure detectors placed at the distances of 0.5, 1, 1.5, and 2 m from the “Qigong” practitioner who emitted “external qi” toward the target through two of his fingers.
	Significant increases in wavelength to above 10 mm during “external qi” emission were measured by mm-wave radiation meter placed 20 to 40 cm from the “Qigong” practitioner.
	Changes were detected in the magnetic field during “qi” emission, in well-controlled conditions in a zero-magnetism laboratory, with signals reaching 105 nT and contrasting with the weak ones emitted by non-practitioners.
	Infrasonic sound pressure measured in acupoints during “qi” emission by experienced practitioners (48.8 to 54.7 dB) were higher than those emitted by the control group (40.6 to 43.6 dB).
Moga&Bengston [25]	Anomalous magnetic field activity was detected during hands-on healing and distant healing adjacent to the mice cages.
Joines et al. [89]	Infrared and ultraviolet light-sensitive equipment detected energies from some healers and meditators who intentionally projected this energy.
Baldwin et al. [94]	No electromagnetic field intensities greater than 3 pT were detected, leading to the conclusion that practicing Reiki does not appear to produce high-intensity electromagnetic fields from the heart or hands routinely.
Moga [90]	Low-frequency magnetic field oscillations were detected during 24 of 26 healing touch sessions and 14 of 16 guided progressive relaxation sessions. The magnetic field oscillations (peak-to-peak) amplitude was significantly greater during the healing touch session and post-session periods than in the pre-session period.

**Table 2:** Research exploring the effects of non-contact biofield practices on biological materials.

Study	Main Findings
Chien et al. [87]	Facilitating “qi” caused a 1.8% increase of the human fibroblast FS-4 growth in 24 h, 10 to 15% increase of Deoxyribonucleic acid (DNA) synthesis, and 3 to 5% increase of protein synthesis of the cell in a 2-h period; inhibiting “qi” caused a 6% decrease of cell growth in a 24 h period, 20 to 23% decrease of DNA synthesis and 35 to 48% of protein synthesis in a 2-h period. The respiration rate of boar sperm increased by 12.5 to 13.0% after 5 min exposure to facilitating “qi” and decreased by 45 to 48% by exposure to 2-min of inhibiting “qi”.
Chu et al. [111]	All poly-d-glutamic acid sodium salt samples presented some changes in circular dichroism spectra measured with a 62DS spectropolarimeter. A total of 67% of those samples had significant changes (more than three standard errors), and a ratio of ellipticity change of 1 to 10% with a maximum change of over 10.9%. No significant changes were noticed on RNA conformation.
Chen [32]	“External qi” was shown to have effects on liver cancer cells (BEL-7402) and lung cancer cell culture (SPC-A), on blood plasma cAMP, on the structure and pharmaceutical characteristics of vitamin C, on the DNA synthesis and living cycles of liver cancer cells, on the phase behaviour of dipalmitoylphosphatidylcholine (DPPC) liposomes, on the microstructure of *Escherichia coli* and tumor cells in mice, on enabling the growth of Fab protein crystals, on inhibiting the growth of hepatitis B virus in vitro.
Yan et al. [97]	The exposure of cultured retinal neurons to “external qi” significantly attenuated neuronal death induced by 24-h exposure to hydrogen peroxide and significantly inhibited hydrogen peroxide-induced apoptosis. “External qi” also upregulated IGF-I gene expression and increased PI3K activity.
Yount et al. [115]	“External qi” increased cell proliferation in normal brain cell samples showing, on average, more colony formation than sham samples (*p* = 0.036); however, in a replication study (60 experiments), no significant difference between treated samples and sham samples was observed (*p* = 0.465).
Taft et al. [116]	No evidence of a reproducible cellular response to “Johrei” treatment was noticed regarding cell death and proliferation rates of cultured human cancer cells.
Yan et al. [96]	“External qi” inhibited basal phosphorylation levels of Akt and extracellular signal-regulated kinases, epidermal growth factor-mediated phosphorylation of extracellular signal-regulated kinases, phosphatidylinositol 3-kinase activity, constitutive and inducible activities of nuclear factor-kappa B. A 5 min exposure of BxPC3 cells to “external qi” induced apoptosis, accompanied by an increase of the sub-G1 cell population, DNA fragmentation, and cleavage of caspases 3, 8 and 9, and poly(Adenosine diphosphate ribose (ADP-ribose)) polymerase. Prolonged exposure caused rapid lysis of BxPC3 cells. Treatment of fibroblasts with “external qi” induced transient activation of extracellular signal-regulated kinases and Akt and caused no cytotoxic effect.
Shao et al. [117]	Opposite intentions of promoting the proliferation of *Escherichia coli* and killing during the “external qi” therapy resulted in higher and lower optical density, respectively, after incubation compared to control.
Yan et al. [118]	“External qi” induced cell death and gene expression alterations, promoting apoptosis and inhibiting proliferation, migration, and glucose metabolism in small-cell lung cancer cells.
Yan et al. [119]	“External qi” decreased viability and blocked colony formation of HT-29 cells, downregulated cyclin D1 expression, and increased the accumulation of cyclin-dependent kinase inhibitors p21(Cip1) and p27(Kip1), resulting in G1 cell cycle arrest. “External qi” induced apoptosis in HT-29 cells in association with decreased expression of anti-apoptotic proteins Bcl-xL, XIAP, survivin, and Mcl-1 and elevated expression of proapoptotic protein Bax. “External qi” significantly repressed phosphorylation of Akt and Erk1/2 and NF-ĸB activation in HT-29 cells, suggesting a cytotoxic effect through regulating signaling pathways critical for cell proliferation and survival.
Lucchetti et al. [120]	Significant differences were found between the Spiritist “passe” and “no laying on of hands (LOH)” groups (*p* = 0.002 after 48 h, and *p* = 0.008 after one week) and also between the Spiritist “passe” and “LOH” groups (*p* = 0.005 after 48 h, and *p* = 0.009 after one week) while inhibiting bacterial growth. No statistically significant difference was found in the experiments with no intention, with an intention to promote growth, and when LOH was performed under the influence of a negative factor.
Trivedi et al. [121]	Biofield treatment exponentially increased (41%) the cell death rate of human glioblastoma, compared to control, which remained relatively constant throughout the 20-h testing period. The treated healthy brain cultured cells showed a significant reduction (64%) of the death rate.
Yan et al. [122]	“External qi” induced apoptosis in A549 cells, resulting in a pronounced reduction in viability and clonogenic formation, associated with inhibition of phosphorylation of Akt and Erk1/2 and reduced expression of anti-apoptotic proteins Bcl-xL, XIAP, and survivin. “External qi” inhibited EGF/EGFR signaling, and EGF mediated migration and invasion of A549 cells. While TGF-β1 induced phosphorylation of SMAD2/3 and EMT in A549 cells, “External qi” suppressed TGF-β/SMAD signaling and induced cell death in these cells in the presence of TGF-β1.
Kent et al. [114]	“Reiki” treatment significantly increased the photon emission of mice intervertebral disc cells compared to sham (*p* < 0.05) and increased collagen II and aggrecan (*p* < 0.05) measured by real-time PCR.

Abbreviations: cyclic adenosine monophosphate (cAMP); insulin like growth factor (IGF-I); phosphoinositide 3-kinase (PI3K); human pancreatic cancer cell line (BxPC3); protein that in humans is encoded by the CCND1 gene (cyclin D1); protein cyclin-dependent kinase inhibitor 1 (p21(Cip1)); protein cyclin-dependent kinase inhibitor 1B (p27(Kip1)); the first phase of the cell cycle that takes place in eukaryotic cell division (G1); human colon cancer cell line (HT-29); B-cell lymphoma-extra large (Bcl-xL); X-linked inhibitor of apoptosis protein (XIAP); protein kinase B (PKB also known as Akt); extracellular signal-regulated kinase 1/2 (Erk1/2); nuclear factor-kB (NF-kB); epidermal growth factor/epidermal growth factor receptor (EGF/EGFR); transforming growth factor beta 1 (TGF-β1); proteins that are the main signal transducers for receptors of the transforming growth factor beta (SMAD2/3); epithelial–mesenchymal transition (EMT).

**Table 3:** Research exploring the effects of non-contact biofield practices on the human body.

Study	Main Findings
Lee et al. [137]	“External qi” therapy induced significant changes accessed by encephalography and circulating cortisol concentrations in the real intervention compared to the placebo control. Subjects reported improved emotions of satisfaction, relaxation, and calmness during the real intervention compared to placebo treatment.
Jang&Lee [138]	Subjects who received “qi” therapy had significant improvements in negative feelings, pain, water retention, and total PMS symptoms, compared to placebo control.
Lee et al. [139,140]	“Qi” therapy produced significant effects in heart rate (HR), low frequency (LF), high frequency (HF), and LF/HF. “Qi” therapy reduced the HR and increased HRV as indicated by a reduced LF/HF power ratio of HRV. “Qi” therapy had a higher capacity to stabilize the sympathovagal function compared to placebo.
Yang et al. [141]	Compared to the control group, the “qi” therapy participants experienced improvements in mood and psychological variables over the four-week program. Pain and psychological benefits remained significantly improved after two weeks of follow-up.
Lee et al. [86]	All RCTs on external “Qigong” demonstrated more significant pain reduction in “Qigong” groups than control groups, including general care for treating chronic pain.
Cohen et al. [142]	No clinical changes were detected in breast cancer tumor measurements from pre- to post- “external qi” therapy, no suggestion of a change in tumor size by physical breast examination, and no changes in quality of life.
Pike et al. [143]	An enhancement of the left-anterior activation of the cerebral cortex relative to placebo and no-treatment controls was seen during the first 100 s of biofield treatment, indicating a higher overall reduction in state anxiety relative to baseline measures.
Gaillard et al. [144]	Non-contact biofield therapy could not reduce the number of warts three weeks after intervention (0/64). No significant differences were found between the experimental and placebo groups concerning warts disappearance three weeks after intervention (*p* = 0.49), nor at six weeks (*p* = 0.40); however, reduction in size at week 3 was more promising for biofield therapy but, even so, was not significant (*p* = 0.27).

Abbreviations: premenstrual syndrome (PMS); heart rate (HR); low frequency (LF); high frequency (HF); low frequency-high frequency ratio (LF/HF); heart rate variability (HRV); randomized controlled trial (RCT).
